# Characterization of *Salmonella enterica* Isolates from Diseased Poultry in Northern China between 2014 and 2018

**DOI:** 10.3390/pathogens9020095

**Published:** 2020-02-04

**Authors:** Jun Wang, Jinxin Li, Fengli Liu, Yongyou Cheng, Jingliang Su

**Affiliations:** 1 Key Laboratory of Animal Epidemiology of the Ministry of Agriculture, College of Veterinary Medicine, China Agricultural University, Beijing 100193, China; luodian825@cau.edu.cn (J.W.); b20193050453@cau.edu.cn (J.L.); bs20193050480@cau.edu.cn (F.L.); 2 College of Food and Pharmaceutical Engineering, Guiyang University, Guiyang City 550002, Guizhou Province, China; chengang01@caas.cn

**Keywords:** *Salmonella*, duck, chicken, pigeon, MLST, PFGE, antimicrobial resistance

## Abstract

*Salmonella* infection not only causes acute and chronic diseases in poultry flocks, but the infected poultry are among the most important reservoirs for a variety of *Salmonella* serovars frequently transmitted to humans. This study aimed to investigate the occurrence of *Salmonella* spp. in local poultry farms in China. Samples (n = 4255), including dead-in-shell embryos, culled day-old-hatchings and 1- to 4-week-old diseased birds, were collected for *Salmonella* culture from broiler chicken, meat-type duck and pigeon farms in northern China between 2014 and 2018. A total of 103 *Salmonella* were isolated. *S.* *enterica* serovar Enteritidis and *S.* Typhimurium were the most prevalent serovars, representing 53.4% and 34.9% of the isolates, respectively. Serovar diversity was the highest in ducks, with the *S.* Apeyeme being isolated for the first time from duck tissues. All isolates were characterized by multilocus sequence typing (MLST) and pulsed-field gel electrophoresis (PFGE). MLST showed that all *S.* Enteritidis isolates shared the same sequence type (ST11), and Typhimurium showed several rare STs in addition to ST19. In comparison, PFGE showed better discrimination for *S.* Enteritidis and *S.* Typhimurium isolates, with nine distinct pulsotypes being observed. The isolates exhibited varying degrees of resistance to 15 tested antimicrobials and identified *S.* Enteritidis isolates (98.18%) with multiple antimicrobial resistance were a cause for concern. Our data on invasive *Salmonella* infection in meat-type poultry in local farms can be used to identify sources and factors associated with *Salmonella* spread in poultry and the associated food chain.

## 1. Introduction

*Salmonella* spp. are one of the leading bacterial pathogens in human beings and domesticated and wild animals [1,2]. It has been estimated that *Salmonella* enterocolitis resulted in 95.1 million cases and 50,771 deaths in 2017 [3,4,5]. In addition to diarrhea, 535,000 cases of nontyphoidal *Salmonella* invasive disease occurred, as well as an estimated 77,500 deaths [6]. Animals are the primary reservoirs of *Salmonella*; especially poultry and poultry products are among the most frequently implicated animal sources for *Salmonella* entering the human food supply. In China, 22.16% of bacterial foodborne illnesses were caused by *Salmonella* between 1994 and 2005 [7]. Contaminated poultry foods including meat and eggs are an important route of transmission of *Salmonella* from animals to humans [8,9]. 

China is a large producer of poultry, especially with the largest production of meat-type ducks and pigeons [10,11]. In contrast to intensively produced broiler chickens, commercial ducks and pigeons are more frequently reared in semi-intensive housing systems using simple accommodations with access to outside pens and bathing water or sands. In practice, *Salmonella* can be introduced to poultry flocks from multiple sources, e.g., from the environment, feed and vectors due to the lack of efficient biosecurity measures [12]. Poultry can become infected with different *Salmonella* serotypes with high morbidity and mortality during the first three weeks of their life, and may also become carriers which can disseminate bacteria spread horizontally within flocks, or vertically to progeny via contaminating breeding eggs, leading to embryo mortality or rapid death of newly hatched birds. The prevalence of *Salmonella* infection in Chinese poultry farms has been widely described [13,14]. In the chicken sector, voluntary control in breeding flocks in combination with national statutory elimination of *S*. Pullorum has reduced *Salmonella* infection. However, ducks and pigeons are not included and limited information on *Salmonella* prevalence is available. Concerning poultry production and food safety, it is important to understand the prevalence of the pathogen in terms of its control.

The aim of the present work was to study the epizootic status of *Salmonella* in the local meat-type poultry farms. Strains isolated from dead-in-shell embryos, culled day-old birds and tissue samples of diseased poultry between 2014 and 2018 were characterized by: (1) serotyping, (2) genotyping using multilocus sequence typing (MLST) and pulsed-field gel electrophoresis (PFGE) and (3) antimicrobial susceptibility testing. 

## 2. Results

### 2.1. Isolation and Serotyping of Salmonella 

Based on the growth characteristics on MacConkey and XLD (Xylose Lysine-Deoxycholate) agar plates, presumptive *Salmonella* colonies were subcultured and screened by PCR based on the *Salmonella*-specific *invA* gene [15]. From 4255 clinical poultry samples, a total of 103 (2.42%) *Salmonella* were isolated (Table 1). Using the classical serotyping agglutination method, these isolates were assigned to eight serotypes distributed in seven O groups. Among these isolates, the highest occurring serotype was *S.* Enteritidis (n = 55), followed by *S.* Typhimurium (n = 36), *S.* Anatum (n = 3), *S.* Apeyeme (n = 3), *S.* Kottbus (n = 2), *S.* Senftenberg (n = 2), *S.* Montevideo (n = 1) and *S.* Pullorum (n = 1). The high frequency of the two serotypes Enteritidis and Typhimurium in the clinical samples is in agreement with their overall prevalence in poultry flocks in the European Union [16]. The distribution of the serotypes varied with host species. For pigeon samples, only serotype Typhimurium was isolated, whereas almost all isolates recovered from chicken were *S.* Enteritidis except for one *S.* Pullorum isolate. The greatest serotype diversity was observed among isolates from duck tissue samples. Of note, *S.* Apeyeme was recovered from duck origin samples for the first time in China. 

### 2.2. Multilocus Sequence Analysis

**Figure 1:**
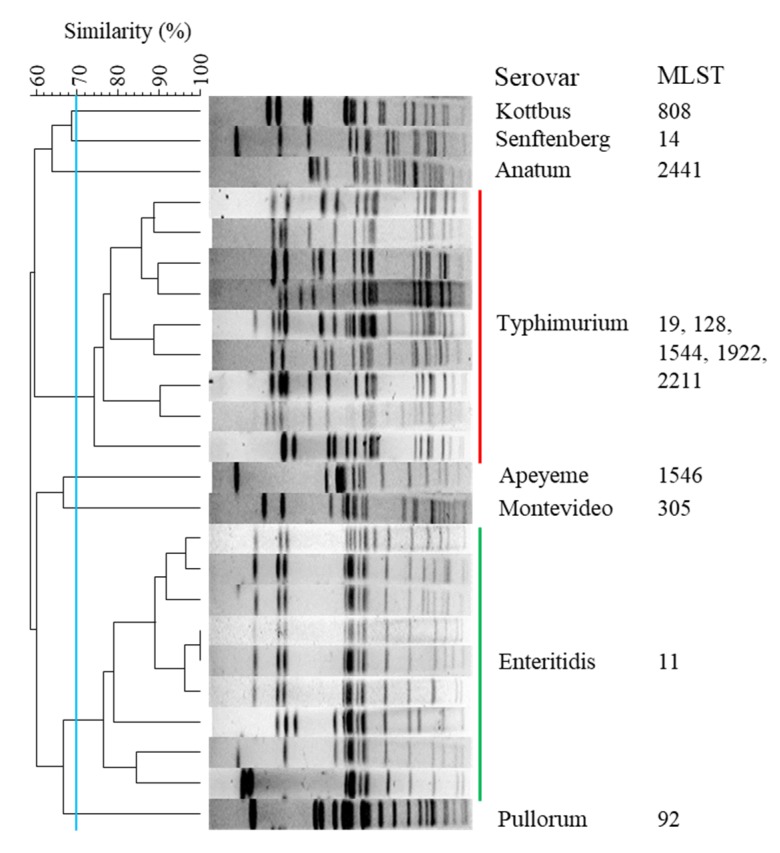
*Xba*I-pulsed-field gel electrophoresis (PFGE) profile and multilocus sequence typing (MLST) of eight *Salmonella* serotypes. A total of 103 isolates are assigned into eight groups with lower than 70% similarity. The isolates of Typhimurium and Enteritidis are grouped into nine profiles with at least one band difference. With the exception of serovar Typhimurium, the remaining serotypes display the same MLST of within-serotypes.

MLST typing based on the comparison of internal sequences of seven housekeeping gene fragments was carried out on the 103 isolates. With exception of *S.* Typhimurium, sequence type assignment exhibited a one-to-one relationship with serovar (Figure 1). All 55 *S.* Enteritidis isolates were assigned to ST11. By contrast, the 36 *S.* Typhimurium isolates were subtyped into five STs, namely ST19 (23/36), ST1922 (7/36), ST1544 (4/36), ST128 (1/36) and ST2211 (1/36). It was noted that the STs represented in our collection were all single-locus variants of ST19, with only one nucleotide difference (Table 2). Interestingly, seven isolates consisting of ST1922 were exclusively isolated from diseased pigeons.

### 2.3. PFGE Analysis 

When the genomic DNA of all *Salmonella* isolates was digested with the *Xba*I enzyme and separated by PFGE, the DNA fragments had a good separation, giving 11 to 19 bands per isolate. The 103 *Salmonella* isolates were successfully assigned into 8 groups representing different serotypes. The genetic similarity among these groups was lower than 70% (Figure 1). 

**Figure 2:**
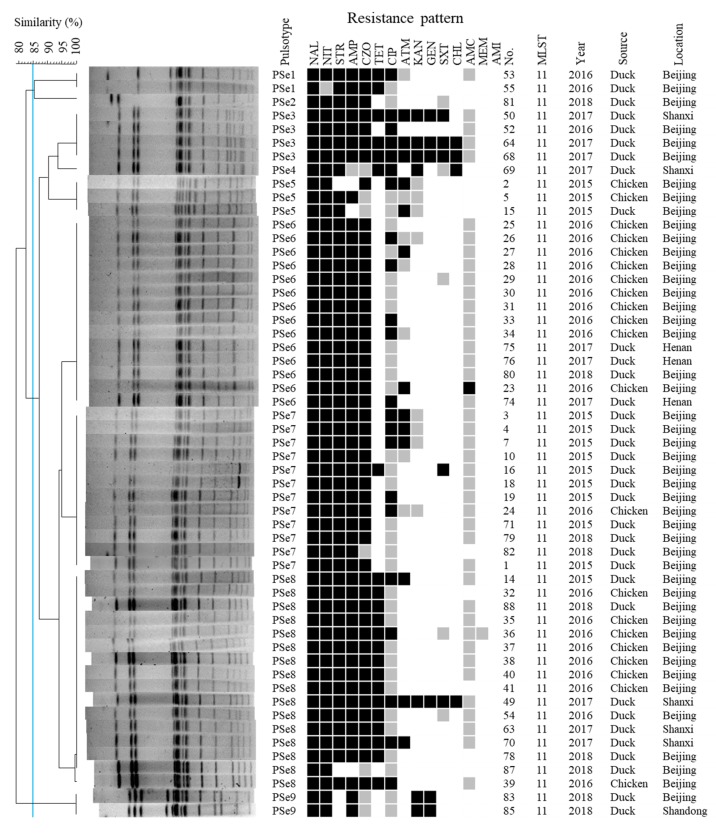
*Xba*I-PFGE profiles, antimicrobial resistance pattern and MLST of all *S.* Enteritidis isolates. For PFGE, 55 isolates were divided into three subtypes according to an 85% similarity. A black box indicates resistance against specified antimicrobial, a gray box indicates intermediate resistance and a white box indicates susceptibility. Antimicrobial designations are detailed in Section 4.

With respect to *S.* Enteritidis, 55 isolates exhibited genetic relatedness ranging from 79.1% to 100%. Defining subtypes as those with at least 85%-similarity banding patterns resulted in 3 subtypes with at least a three bands difference. All 55 *S.* Enteritidis could be grouped within three main PFGE subtypes at an 85% genetic similarity (Figure 2). For further visual analyses based on the dendrogram, isolates were regarded as having a distinct PFGE profile if they differed by at least one band from other isolates. By these criteria, these isolates were primarily grouped into nine pulsotypes (labeled PSe1 through PSe9) (Figure 2) with within-profile similarity ≥ 99.5%. PSe 1, 2, 3, 4 and 9 were consisted by duck isolates. The profiles PSe 5, 6, 7 and 8 were observed in isolates from chicken and duck samples, containing a majority of our *S.* enteritidis isolates (duck = 23, chicken = 19). It was evident that PFGE analysis yielded a greater differentiation than that of the MLST on the *S.* Enteritidis subtyping in this study. 

**Figure 3:**
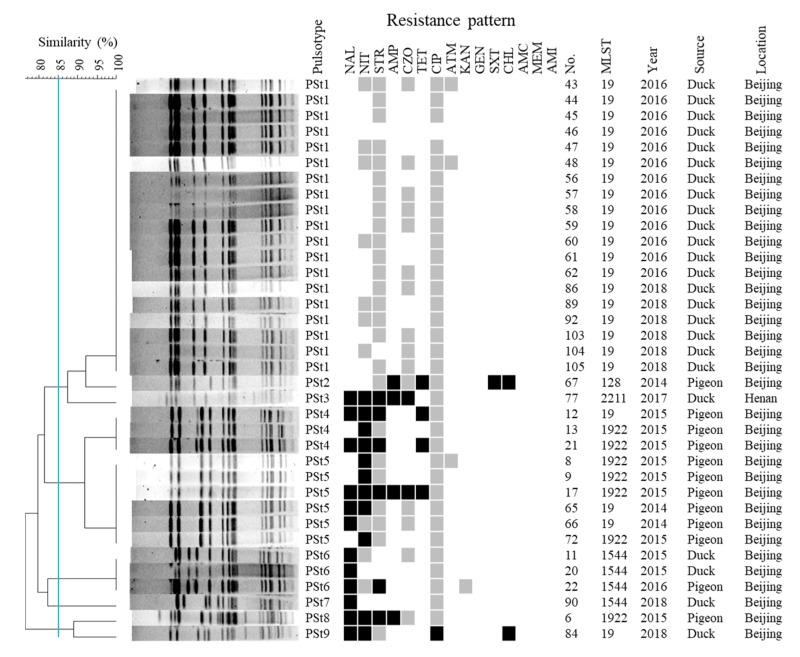
XbaⅠ-PFGE profiles, antimicrobial resistance pattern and MLST of all *S*. Typhimurium isolates. For PFGE, 36 isolates were divided into three subtypes according to an 85% similarity. A black box denotes resistance against specified antimicrobial, a gray box denotes intermediate resistance and a white box denotes susceptibility. Antimicrobial designations are detailed in Section 4.

Using the same criteria, the 36 *S.* Typhimurium isolates were grouped into five subtypes (> 3 bands difference) after *Xba*I digestion, and further divided into nine PFGE profiles (PSt1 through PSt9) with ≥ 1 band difference) (Figure 3). We noted that isolates from ducks and pigeons exhibited distinct banding profiles except for one isolate from pigeon that was assigned to PSt6 with two duck-origin isolates. Most duck isolates (19/20) that were assigned to ST19 by MLST typing were grouped in PSt1 except one, which was grouped into PSt9. PSt6 contained two duck- and one pigeon-origin ST1544 isolates, and one duck-origin ST1544 isolate displayed a distinct banding profile (PSt7). The only Typhimurium isolated from a duck sample in Henan was assigned as ST2211, exhibiting a different banding pattern (PSt3). Most pigeon isolates distributed in PSt5 (6/12) and PSt4 (3/12) with only one band difference between them. In general, *Xba*I PFGE generated a higher index of discrimination for both *S.* Enteritidis and *S.* Typhimurium. 

### 2.4. Salmonella Isolates Displayed High Levels of Antimicrobial Resistance 

The 103 tissue-culture *Salmonella* isolates were tested for susceptibility to 15 antimicrobial drugs. Resistance to at least one drug was observed among 77 isolates (74.8%), representing ten different resistance patterns (Table 3). Although no resistance was observed for the other 26 isolates, 25 of them exhibited intermediate resistance to 1 to 4 drugs, including meropenem (MEM). One isolate exhibited pan-susceptibility to all tested antimicrobials. 

When stratified by serotype, *S.* Enteritidis displayed a significantly higher frequency of antimicrobial resistance than that of other serotypes. Except isolate 87, all *S.* Enteritidis isolates (n = 54) were resistant to ≥ 3 antimicrobial classes, which is considered multidrug resistance (MDR) [17]. The most frequent resistance phenotypes were nalidixic acid (NAL) (100%), nitrofurantoin (NIT) (98.2%), streptomycin (STR) (92.7%), ampicillin (AMP) (92.7%) and cefazolin (CZO) (87.3%), followed by tetracycline (TET) (40%), aztreonam (ATM) (23.6%), ciprofloxacin (CIP) (22.33%), kanamycin (KAN) (12.7%), gentamicin (GEN) (10.9%), trimethoprim/sulfamethoxazole (SXT) (9.1%), chloramphenicol (CHL) (7.3%) and amoxicillin/clavulanic acid (AMC) (1.8%). The most common pattern of resistance involved NAL-NIT-STR-AMP-CZO and NAL-NIT-STR-AMP-CZO-TET, with 14 and 12 isolates, respectively. Association relationship between species and resistance patterns was not significantly observed. However, four isolates recovered from ducks in 2017 displayed resistance to 11 to 12 tested antimicrobials.

Compared with the collection of *S.* Enteritidis, *S.* Typhimurium isolates exhibited a significantly lower frequency of antimicrobial resistance. 17 of the 36 isolates were resistant to at least one antimicrobial. Antimicrobial resistance was predominantly observed in pigeon-origin isolates. All pigeon isolates (12/12) were resistant to at least one of the tested antimicrobials and five of them exhibited multidrug resistance (≥ 3 antimicrobial classes). By contrast, five duck isolates (5/24) had antimicrobial resistance and two were resistant to three (NAL-NIT-CHL) and five (NAL-NIT-STR-AMP-CZO) antimicrobials.

## 3. Discussion

In this study, we analyze 103 *Salmonella* isolates from poultry tissue with clinical disease, which provides relevant results to understand the epidemiology of *Salmonella* infection in meat-type poultry farms. Invasive *Salmonella* isolates involving the death of hatching embryos, hatched-duck culling, fatal infection of table ducklings and broiler chickens in parts of northern China, between 2014 and 2018, were typified at the serovar level and by an approach involving several molecular typing methods. The serovars identified in this study indicate the diversity of *Salmonella* in commercial poultry flocks in northern China. *S.* Enteritidis and *S.* Typhimurium were the most frequently isolated, consistent with previous reports from China and some European countries [18,19]. Of the remaining isolates, *S.* Anatum, *S.* Senftenberg, *S.* Kottbus and *S.* Montevideo were also commonly isolated from poultry-based products and humans in China [20,21,22]. This result reflects the common occurrence of these serovars in meat-type poultry, highlighting the reservoirs being maintained in the poultry practices. It is notable that *S.* Apeyeme was isolated from clinical poultry samples for the first time. Although the pathogenicity of *S.* Apeyeme needs further investigation, its emergence as a zoonotic pathogen is of concern because it has been isolated from human, shellfish and apple snail (https://enterobase.warwick.ac.uk/species/index/s*enterica*).

In the present study, 74 (71.84%) *Salmonella* strains were isolated from tissues of diseased poultry, highlighting the association with clinical infection on these poultry farms. Remarkably, the majority of these isolates distributed in diseased duck (59, 57.28%) and pigeon tissues (12, 11.65%). The relatively high frequency of invasive infection of *Salmonella* in commercial meat-type duck and pigeon flocks may largely relate to semi-open rearing systems that lack effective biosecurity. Birds are reared on the ground or on slatted plastic flooring, whereby *Salmonella* can be introduced into flocks from many sources and transmitted efficiently within a flock. Isolation of seven serovars from diseased duck samples also indicated a high level of *Salmonella* diversity in commercial duck flocks as in a previous study [23]. Duck might serve as a reservoir of various *Salmonella* serotypes. It has been reported that infection of ducklings most likely happens at the hatchery [24]; however, the isolation rate of *Salmonella* spp. from dead-in-shell embryos and culled 1-day-old ducklings in our study was 0.41% (10/2428), which was significantly lower than those reported in previous studies (2.1% and 12.2%) [13,23]. This difference might be influenced by the environmental conditions on the sampled farms, where breeding eggs were fully washed and sanitized with chlorine bleach before entering the hatchery. By contrast, 1-day-old chicks were found to have a *Salmonella* prevalence of 4.2% (19/450). This high prevalence might be in relation to sample collections. These samples were targeted for *Salmonella* because broiler chickens derived from these hatcheries were experiencing *Salmonella* infection during the test period. 

From the diseased meat-type pigeon samples, only *S.* Typhimurium was identified in this study, and it remains to be seen whether these isolates belong to a host-adapted serovar as reported [25,26]. However, care should be taken when interpreting serotype data from broiler chickens and pigeons because all pigeon Typhimurium isolates originated from dead pigeons submitted for clinical diagnosis, and may not, therefore, represent the true distribution of the serotypes in these populations. Previous reports have revealed that five serovars (Typhimurium, Enteritidis, Derby, Brandenburg and Pomona) were recovered from meat, liver, intestine and cloacal swab of pigeons in China, Madrid and Flanders [27,28,29]. As for the distribution of *Salmonella* serotypes in three chicken farms, only *S*. Enteritidis and *S*. Pullorum assigned into the same O group were isolated. Along with the result, slide agglutination tests of 143 sera collected from these farms were performed using inactivated *S*. Pullorum antigen. 40 (27.97%) sera showed positive reaction. This result was consistent with *Salmonella* isolation.

MLST and PFGE have been widely used to subtype *Salmonella* isolates for genetic relatedness and epidemiological evaluation. In this study, both MLST and PFGE subtyping grouped isolates together into the representing serovar. All 55 Enteritidis isolates were assigned into ST11, a vastly distributed clone across the world [14,30,31]. However, PFGE analysis showed that the 55 isolates displayed nine banding patterns distinguishable by at least one band difference (Figure 2); this banding pattern similarity was higher than that of *S.* Enteritidis isolates from breeder chickens reported recently [32]. No obviously epidemiological relatedness was explored from the same and different samples. Although the vast majority of Enteritidis isolates were grouped in subtype E2, the distinct patterns of isolates in subtypes E1 and E3 indicated that PFGE provided greater discrimination for subtyping *S.* Enteritidis than did MLST [33].

In this study, the majority of *S.* Typhimurium isolates were grouped into ST19 (63.89%), the most common sequence types isolated from humans and animal-based food products across the world [34,35]. Moreover, ST128, ST1544 and ST2211 have been identified from human and animal samples in China and the United Kingdom [28,36]. Perhaps circulatory transmission generated between contaminated poultry meat and human beings. Interestingly, the ST1922 in our collection consisted of seven isolates originating exclusively from diseased pigeons. A previous study has revealed that ST1922 Typhimurium was originally isolated from liver of pigeon sold in one market in Sanya, China [37]. To our knowledge, ST1922 of Typhimurium has been specifically isolated from pigeon samples until now. Further analysis may be needed to explore the restrictive relationship between the ST type and pigeon host. Besides, other ST types of Typhimurium were also recovered from meat of pigeon recently [28]. Using PFGE subtyping the 36 Typhimurium isolates were classified into five subtypes with a < 85% similarity (Figure 3). Most of the duck- and pigeon-origin Typhimurium isolates were distributed in subtype 1 and subtype 2, respectively, suggesting some association between PFGE and host-origin. On the other hand, 19 *S.* Typhimurium isolates shared identical PFGE patterns in PSt1, suggesting the circulation of clonality among the *S.* Typhimurium in the local duck farms.

The emergence and development of antimicrobial resistance of *Salmonella* isolates from animals and their products may be due to a variety of reasons [38]. The qualitative results determined by the disk diffusion method suggested that *Salmonella* isolates in our collection exhibited varying resistance to the 15 tested antimicrobials. It should be noted that antibiotics are frequently used on poultry farms, which may be the cause of the resistance to the tested antibiotics. In this work, high rates of resistance to nalidixic acid (65.05%), nitrofurantoin (63.11%), streptomycin (55.34%), ampicillin (53.40%) and cefazolin (48.54%) were observed. This result agreed with previous reports in China, Iran and the United States [28,39,40]. Beyond that, high resistance of Enteritidis has been reported frequently [39,41], but 100% (55/55) resistance was somewhat unexpected, especially the extremely high frequency (54/55) of MDR. These agents containing nalidixic acid, nitrofurantoin, streptomycin, ampicillin and cefazolin have been extensively used in poultry practices in China, which was noted by the Chinese veterinary pharmacopoeia and the report [42]. High prevalence of antimicrobial resistance should draw our attention. Further studies are needed to assess factors that might play a role in the development of AMR (antimicrobial resistance) in commercial poultry practices in northern China. However, our study might have overestimated the frequency of AMR because some samples came from birds that might already have been treated with antimicrobials. *S.* Typhimurium in general exhibited decreased resistance to most of the 15 antimicrobials. This result was inconsistent with other reports in China [43,44]. Nevertheless, more resistant isolates were found in diseased pigeon samples than in duck-origin ones. We speculate that the frequency of utilization and types of agents were quite different in these farms through clinical interrogation, which is supported by the study. [19].

In conclusion, our study showed the presence of *Salmonella* serovars in clinically diseased birds in local poultry flocks. High frequency isolation of the most common serovars Enteritidis and Typhimurium indicates that meat-type poultry could be reservoirs for zoonotic *Salmonella* strains. The extremely high rate of antimicrobial resistance in clinical *Salmonella* Enteritidis isolates is of great concern for antimicrobial use in poultry practice, and may pose a threat to human health. The observed association between the serotypes and molecular subtypes of *Salmonella* isolates in this study suggest that combining results obtained from all three methods might provide more useful epidemiological information related to infection. 

## 4. Materials and Methods 

### 4.1. Salmonella Isolation and Serotyping

Isolation and identification of *Salmonella* were conducted following the OIE (World Organisation for Animal Health) manual for salmonellosis diagnostic test with partial modifications. One- to four-week-old ducks, broiler chickens and pigeons from poultry farms distributed in Henan, Shanxi, Shandong, Hebei and Beijing from 2014 to 2018 were sampled for diagnosis. For birds with observable gross lesions (e.g., perihepatitis and swollen liver at autopsy), the surface of liver was branded with scalding surgical blade and the skull was disinfected with 75% ethanol and opened using sterile scissor. Disinfected tissue was then streaked onto MacConkey agar plates and tryptic soy agar plates (Becton, Dickinson and Co, Sparks, MD, USA) by inoculation loop. After incubation at 37 °C for 18 to 24 h, suspected colonies from every plate were picked and subcultured on MacConkey plates. For dead-in-shell embryos and 1-day-old hatchings culled at the hatchery, the whole liver and cecum of every one were aseptically obtained and sliced, pooled (in groups of 5) and placed into 100 mL of buffered peptone water (BPW) (Beijing Landbridge Technology Co., Ltd., Beijing, China). Following incubation at 37 °C for 24 h, 1 mL of culture was transferred into 10 mL of Rappaport–Vassiliadis enrichment broth and incubated at 37 °C for 24 h. Then one loop of the broth culture was streaked onto XyloseLysine-Deoxycholate Agar (XLD) plate (Becton, Dickinson and Co, Sparks, MD, USA) and characteristic colonies were picked and subcultured after incubation at 37 °C for 24 h. To screen for *Salmonella*, the suspected colonies were screened by PCR assay with the primer pair (5′-GTGAAATTATCGCCACGTTCGGGCAA-3′/5′-TCATCGCACCGTCAAAGGAACC-3′) based on the *Salmonella invA* gene. Selected *Salmonella* were subcultured and serotyped by slide agglutination using commercial O and H antisera to distinguish *Salmonella* serovars (Tianrun Bio-Pharmaceutical, Ningbo, China) in accordance with the White–Kauffmann–Le Minor scheme [45]. All identified isolates were stored at −80 °C for further use. 

### 4.2. Multilocus Sequence Typing

MLST for the seven housekeeping genes, including *thrA*, *purE*, *sucA*, *hisD*, *aroC*, *hemD* and *dnaN* was performed according to protocols described previously [46]. Briefly, all strains stored at -80 °C were streaked onto tryptic soy agar and incubated at 37 °C for 12 h; then one colony was picked and inoculated into tryptic soy broth. After overnight incubation, 50 μl of bacterial culture was collected and boiled at 95 °C for 10 min. The suspension was briefly centrifuged to remove cellular debris and the supernatant was used as a DNA template for the PCR assay. All PCR amplification was performed in a volume of 50 μl containing 25 μl of 2 × Taq PCR Mix (GenStar, Beijing, China), 4 μl of template, 2 μl of each 25 μM primer and 17 μl of sterile ddH_2_O. The PCR reactions were conducted with the following thermal cycling profile: initial denaturation at 94 °C for 5 min followed by 30 cycles of 94 °C for 30 s, 56 °C for 30 s, 72 °C for 30 s and an additional extension at 72 °C for 7 min. The PCR products were gel-purified and subjected to bidirectional DNA sequencing using an Applied Biosystems ABI3730 sequencer (Meiji Biological Medicine Technology Co., Ltd. Shanghai, China). Sequences were aligned using Megalign (Lasergene 10, DNAStar, Madison, WI, USA). The nucleotide sequence for each gene fragment was submitted to the *Salmonella* MLST database (http://mlst.warwick.ac.uk/mlst/dbs/Senterica) to obtain the specific *Salmonella* sequence type. 

### 4.3. Pulsed-field Gel Electrophoresis 

PFGE of 103 isolates was performed following the PulseNet protocol with modification [47]. Briefly, 200 μl of the overnight culture of each isolate was added to agarose for plug preparation. Plugs were lysed at 54 °C for 2 h, and the obtained DNA was digested with 50 U of *Xba*I (TaKaRa, Dalian, China) at 37 °C for 4 h. The restriction fragments were separated in a 0.5 × Tris-borate-EDTA buffer at 14 °C for 18 h using a Chef Mapper electrophoresis system (Bio-Rad Laboratories, Hercules, CA, USA) with switch times of 2.16 s and 63.8 s. The gels were stained with GoldenView and visualized under UV transillumination. *Salmonella* Braenderup H9812 was used as the standard molecular marker. Images were analyzed using the InfoQuest software version 4.5 (Bio-Rad Laboratories). The PFGE patterns were analyzed according to the Dice similarity coefficient method using the BioNumerics software (version 7.6; Applied Maths, Kortrijk, Belgium). The unweighted pair group method with arithmetic mean (UPGMA) was selected to construct a dendrogram with a position tolerance of 1.5% and optimization of 1.5%.

### 4.4. Antimicrobial Resistance Test

The susceptibility of 103 isolates to a panel of 15 antimicrobials was determined using the Kirby–Bauer disk diffusion method according to the Clinical and Laboratory Standards Institute (CLSI) recommendation [48]. The antimicrobials and corresponding concentration included ampicillin (AMP, 10 μg), amoxicillin/clavulanic acid (AMC, 20/10 μg), cefazolin (CZO, 30 μg), meropenem (MEM, 10 μg), aztreonam (ATM, 30 μg), kanamycin (KAN, 30 μg), gentamicin (GEN, 10 μg), streptomycin (STR, 10 μg), amikacin (AMI, 30 μg), tetracycline (TET, 30 μg), trimethoprim/sulfamethoxazole (SXT, 1.25/23.75 μg), ciprofloxacin (CIP, 5 μg), nalidixic acid (NAL, 30 μg), chloramphenicol (CHL, 30 μg) and nitrofurantoin (NIT, 300 μg). *Escherichia coli* ATCC25922 was used as the quality control.

## Figures and Tables

**Table 1:** *Salmonella* identification of diseased birds.

Serovar	Duck			Chicken		Pigeon	Total
	Dead-in-Shell Embryo (n = 1028)	Day-old Hatching (n = 1400)	One- to Four-Week-Old Duck (n = 1307)	Dead-in-Shell Embryo (n = 450)	One- to Four-Week-Old Chicken (n = 15)	Liver or Brain of Dead Pigeon (n = 55)	4255
**Enteritidis**	1	-	33	19	2	-	55
**Typhimurium**	2	-	22	-	-	12	36
**Apeyeme**	-	3	-	-	-	-	3
**Anatum**	2	1	-	-	-	-	3
**Senftenberg**	-	-	2	-	-	-	2
**Kottbus**	-	-	2	-	-	-	2
**Montevideo**	-	1	-	-	-	-	1
**Pullorum**	-	-	-	-	1	-	1

**Table 2:** Nucleotide discrimination of *S*. Typhimurium MLST.

ST of *S*. Typhimurium	*hisD*		*purE*	*sucA*
19	482 G	539 G	81 T	478 G
128	-	-	-	478 C
1544	-	539 A	-	-
1922	482 A	-	-	-
2211	-	-	81 C	-

**Table 3:** Antimicrobial resistance pattern of 103 *Salmonella* isolates.

Antimicrobial Resistance Patterns	Isolates no.
Susceptible to all tested antimicrobials	26
NAL	7
NIT	4
NAL-STR	1
NAL-TET	1
NAL-NIT	2
NAL-STR-KAN	1
NAL-NIT-CHL	1
NAL-NIT-STR-ATM	1
NAL-NIT-STR-AMP	2
NAL-NIT-STR-ATM	1
AMP-TET-SXT-CHL	1
NAL-NIT-TET-STR	2
NAL-NIT-CIP-CHL	1
NAL-NIT-CZO-CIP-ATM	1
NAL-STR-AMP-CZO-TET	1
NAL-NIT- STR-AMP-CZO	14
NAL-NIT-AMP-KAN-GEN	2
NAL-NIT-STR-AMP-CZO-CIP	8
NAL-NIT-STR-AMP-CZO-TET	12
NAL-NIT-STR-AMP-CZO-CIP-ATM	3
NAL-NIT-STR-AMP-CZO-TET-SXT	1
NAL-NIT-STR-AMP-CZO-ATM-AMC	1
NAL-NIT-STR-AMP-TET-CIP-FAZ	3
NAL-NIT-STR-TET-CIP-KAN-CHL	1
NAL-NIT-STR-AMP-CZO-TET-CIP-ATM	2
NAL-NIT-STR-AMP-CZO-TET-CIP-ATM-KAN-GEN-SXT	1
NAL-NIT-STR-AMP-CZO-TET-CIP-ATM-KAN-GEN-SXT-CHL	3

NAL: nalidixic acid; NIT: nitrofurantoin; STR: streptomycin; AMP: ampicillin; CZO: cefazolin; TET: tetracycline; CIP: ciprofloxacin; ATM: aztreonam; KAN: kanamycin; GEN: gentamicin; SXT: trimethoprim/sulfamethoxazole; CHL: chloramphenicol; AMC: amoxicillin/clavulanic acid; MEM: meropenem; AMI: meropenem.
